# COQ8B glomerular nephropathy: Outcomes after kidney transplantation and analysis of characteristics in Chinese population

**DOI:** 10.3389/fped.2022.938863

**Published:** 2022-08-10

**Authors:** Shuhan Zeng, Yuanyuan Xu, Cheng Cheng, Nannan Yu, Longshan Liu, Ying Mo, Lizhi Chen, Xiaoyun Jiang

**Affiliations:** ^1^Department of Pediatric Nephrology and Rheumatology, The First Affiliated Hospital of Sun Yat-sen University, Guangzhou, China; ^2^Organ Transplant Center, The First Affiliated Hospital of Sun Yat-sen University, Guangzhou, China

**Keywords:** COQ8B, steroid-resistant nephrotic syndrome, kidney transplantation, Chinese, case report

## Abstract

**Background:**

Mutation in the *COQ8B gene* can cause COQ8B glomerular nephropathy (COQ8B-GN), which is rare and associated with steroid-resistant nephrotic syndrome (SRNS) as well as rapid progression to end-stage renal disease (ESRD). The aim of this study was to analyze the prognosis and recurrence risk of COQ8B-GN in patients after kidney transplantation (KTx) and summarize the characteristics of the Chinese population.

**Methods:**

A retrospective study included four cases treated in our hospital with a diagnosis of COQ8B-GN. Chinese and foreign studies were searched from database inception to February 2022.

**Results:**

A total of four cases were included, with the age of onset ranging from 4 to 9 years. The initial presentations were SRNS and asymptomatic proteinuria. Only one had an extrarenal manifestation (thyroid cyst). All patients progressed to ESRD at a mean time of 42 months after onset. With a total follow-up time ranging from 12 to 87 months, three of them had received transplantation. While one case needed a second KTx due to graft failure caused by chronic rejection, two recipients had excellent graft function. No recurrence in allograft was observed. There have been 18 cases of KTx recipients reported globally with follow-up information. Except for two cases of graft failure caused by hyperacute rejection and chronic rejection, respectively, the rest all had good graft function without recurrence. In addition, 44 cases of COQ8B-GN in the Chinese population were identified. At the onset, 75% of the patients were aged ≤10 years with initial symptoms of asymptomatic proteinuria, nephrotic syndrome (NS), or SRNS. By the time of literature publication, 59% of patients had progressed to ESRD (mean age of 10.3 ± 3.6 years). The median time from onset to ESRD was 21 months. Renal pathology mainly showed focal segmental glomerulosclerosis (FSGS), accounting for 61.8% of all biopsies, followed by mesangial proliferative glomerulonephritis (20.6%). The first three prevalent mutations in the *COQ8B gene* among the Chinese population were c. 748G>C, c. 737G>A, and c. 532C>T.

**Conclusion:**

COQ8B-GN in the Chinese population may present with asymptomatic proteinuria, NS, or SRNS initially, with most onsets before the age of 10 years. A lot of patients progress to ESRD in early adolescence. FSGS on biopsy and c. 748G>C in the genetic test are the most frequently seen in Chinese COQ8B-GN patients. KTx is feasible for patients with ESRD due to the low risk of recurrence, but we should pay attention to graft rejection.

## Introduction

COQ8B, also known as aarF domain-containing kinase 4 (ADCK4), is enriched in podocytes and localized to podocyte mitochondria as well as foot processes. It can interact with COQ6 and COQ7, which participate in the biosynthesis of Coenzyme Q10 (CoQ10) (1). CoQ10 (ubiquinone) exists in all cell membranes as a lipid-soluble molecule and plays important roles in antioxidation, pyrimidine synthesis, fatty-acid beta-oxidation activity, and electron transfer of the respiratory chain (2). Thus, *COQ8B gene mutations* can cause primary CoQ10 deficiency and glomerular podocyte injury resulting in COQ8B glomerular nephropathy (COQ8B-GN) or nephrotic syndrome type 9 (NPHS9), which commonly manifests as a steroid-resistant nephrotic syndrome (SRNS) (1). Since podocyte metabolism relies on anaerobic glycolysis, damage to podocytes caused by primary CoQ10 deficiency may result from increased reactive oxygen species and impaired pyrimidine metabolism rather than the loss of oxidative phosphorylation in mitochondria (3). Proteinuria could be relieved in some patients with CoQ10 supplementation (4, 5), especially in those who are asymptomatic or without irreversible renal damage. However, patients with COQ8B-GN often present with insidious onset and rapid progression to end-stage renal disease (ESRD), making the early recognition of the disease difficult. A previous multicenter study (6) has shown that *COQ8B* is the most common causative gene in children with SRNS in China. Nevertheless, report on this disease remains scarce. Current reports mainly focus on non-Chinese cases, and follow-up data after kidney transplantation (KTx) is lacking. In this study, we reported four children with *COQ8B* gene mutation-associated ESRD and conducted a literature review. We analyzed the recurrence risk and prognosis of patients with COQ8B-GN after KTx and summarized the clinicopathological characteristics of COQ8B-GN in the Chinese population for the first time. The disease courses among Chinese cases were compared with that in the non-Chinese population to further improve the understanding of this disease.

## Methods

This study retrospectively collected and analyzed the clinical data, family history, and genetic information from four patients with COQ8B-GN who were treated and followed at the First Affiliated Hospital of Sun Yat-sen University from October 2017 to March 2022. The estimated glomerular filtration rate (eGFR) was calculated using the Schwartz formula (7). ESRD was defined as GFR <15 ml/min/1.73 m^2^ (8). Short stature was defined as a height (Ht) below the third percentile or −1.88 standard deviation (SD) (9). Graft failure referred to a return to dialysis or the requirement of a second KTx (10). Renal allograft rejection was divided into acute rejection that occurred within days/weeks or up to 3 months after KTx, and chronic rejection that occurred 3 months after KTx and presented with progressive loss of graft function (11).

A comprehensive reference from China National Knowledge Infrastructure (CNKI), China WanFang, and foreign studies from PubMed were searched from inception to February 2022. The core was to identify information regarding post-transplantation and clinical data on *COQ8B mutation* in the Chinese population. Two authors conducted the literature search independently using keywords such as *COQ8B, ADCK4*, or nephrotic syndrome type 9. All abstracts of retrieved articles were reviewed, and the selected references were carefully evaluated to identify relevant information. The extracted data included clinical manifestation, genetic testing, pathological biopsy, and patients' outcome when available. Those studies without specific clinical manifestations or follow-up information were excluded.

The study was approved by the First Affiliated Hospital of Sun Yat-sen University. Written informed consent was obtained from the parents of the four patients included.

## Statistical analysis

The data were processed using SPSS 25.0 and Graphpad Prism 9. Continuous variables with normal or non-normal distribution were presented as mean ± SD or median (interquartile interval, IQR), respectively. Data were analyzed using the Mann–Whitney *U*-test or one-way ANOVA. Categorical variables were reported as percentage (%). A *P*-value of <0.05 was considered to be statistically significant.

## Results

### Case series of COQ8B-GN

Case 1 was a 4-year-old girl who initially presented with dysuria and proteinuria but took self-medication without regular treatment and follow-up. The patient was admitted to our hospital on 29 October 2017, with the chief complaint of oliguria and edema. Physical examination revealed high blood pressure (144/113 mmHg), short stature (−2.7 SD), pale and facial, and eyelid and lower limb edema. Laboratory findings showed moderate anemia (hemoglobin, Hb 72 g/L), urinary protein 3+, and hypoalbuminemia (albumin, ALB 29 g/L). Evaluation of renal function demonstrated ESRD (blood urea nitrogen, BUN 59.1 mmol/L; serum creatinine, sCr 1,427 μmol/L; eGRF <5 ml/min/1.73 m^2^). Renal ultrasound revealed bilateral kidney volume shrinkage (left side 6.9 cm × 2.9 cm, parenchyma thickness 1 cm; right side 6.9 cm × 3.1 cm, parenchyma thickness 0.9 cm). Measurement of the glomerular filtration rate (GFR) by renal dynamic imaging also showed a severe decline in both renal functions (GFR: left side 4.3 ml/min, right side 3.3 ml/min). Echocardiography showed left atrial and ventricular enlargement as well as pericardial effusion (trace), which might be caused by uremia. A renal biopsy was not performed due to kidney shrinkage. Whole exome sequencing (WES) analysis was performed after admission, and a homozygous variation in the *COQ8B* gene was detected. The patient started maintenance hemodialysis from 30 October 2017 to 10 April 2018. She then underwent KTx and received an immunosuppressive regimen of mycophenolate mofetil, tacrolimus, and prednisone after the operation. There was no recurrence during 46 months of follow-up with a normal urinary protein level and sCr at the last visit (Table 1).

**Table 1:** Clinical manifestation, genotype, and follow-up data of four cases with COQ8B-GN.

**Patient**	**Gender**	**Onset (years)**	**Initial presentation**	**Extrarenal manifestations**	**Age at ESRD (years)**	**Duration from onset to ESRD (months)**	**Nucleotide alteration (amino acid change)**	**Age at KTx (years)**	**Follow-up period (months)**	**Renal outcome**
No.1	F	4	Dysuria, P	Short stature (−2.7SD)	10	72	c.748G>C HOM (p.D250H)	10.7	45	CKD 1T Negative urinary protein
No.2	F	6	SRNS	No	7	12	c.748G>C HOM	12 (first KTx)	64	Graft loss due to CR
							(p.D250H)	17 (second KTx)	27	CKD 1T Negative urinary protein
No.3	F	9	SRNS	Thyroid cyst Short stature (< −3SD)	15	72	c.748G>C HOM (p.D250H)	15	12	CKD 1T Negative urinary protein
No.4	M	8	P, HBP	No	9	12	c.748G>C (p.D250H) Het c.448C>T(p.R150*,395)Het	-	-	ESRD

F, female; SRNS, steroid-resistant nephrotic syndrome; HOM, homozygous; Het, heterozygous; KTx, kidney transplantation; SD, standard deviation; CR, chronic rejection; P, proteinuria; HBP, high blood pressure; ESRD, end-stage renal disease.

**Figure 1:**
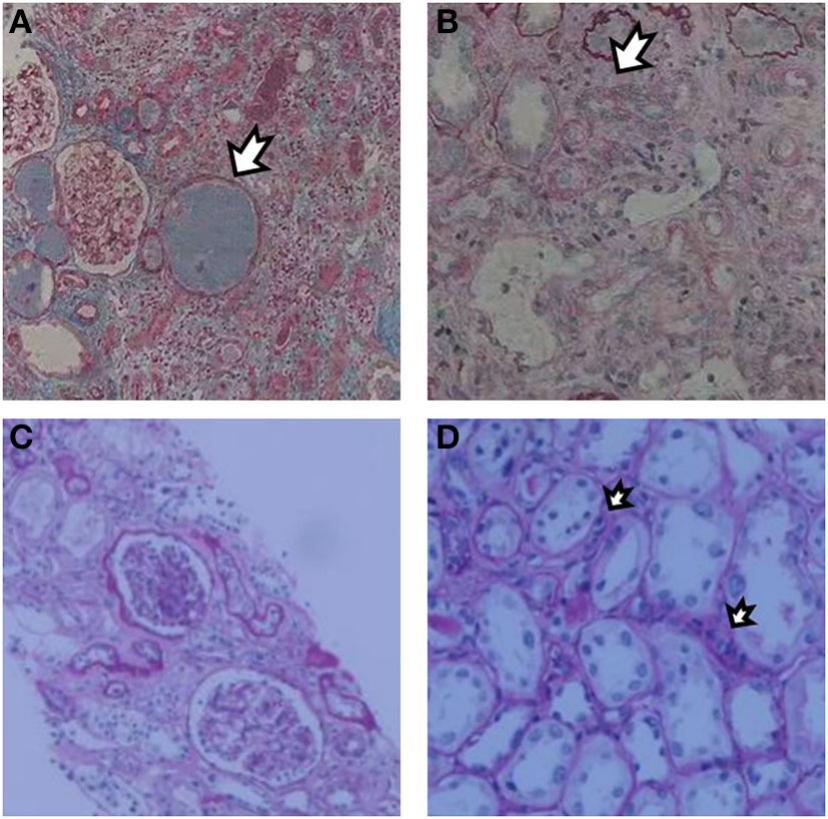
Renal biopsy after the first KTx showed increased formation of protein casts **(A)** with moderate to severe tubular atrophy and diffuse fibrosis of renal interstitium **(B)**. Renal biopsy after the second KTx showed mild proliferation of glomerular mesangial cells and matrix without spherical or segmental sclerosis. Glomerulitis [g1, **(C)**] and peritubular capillaritis [ptc1, **(D)**] were present under a light microscope.

**Figure 2:**
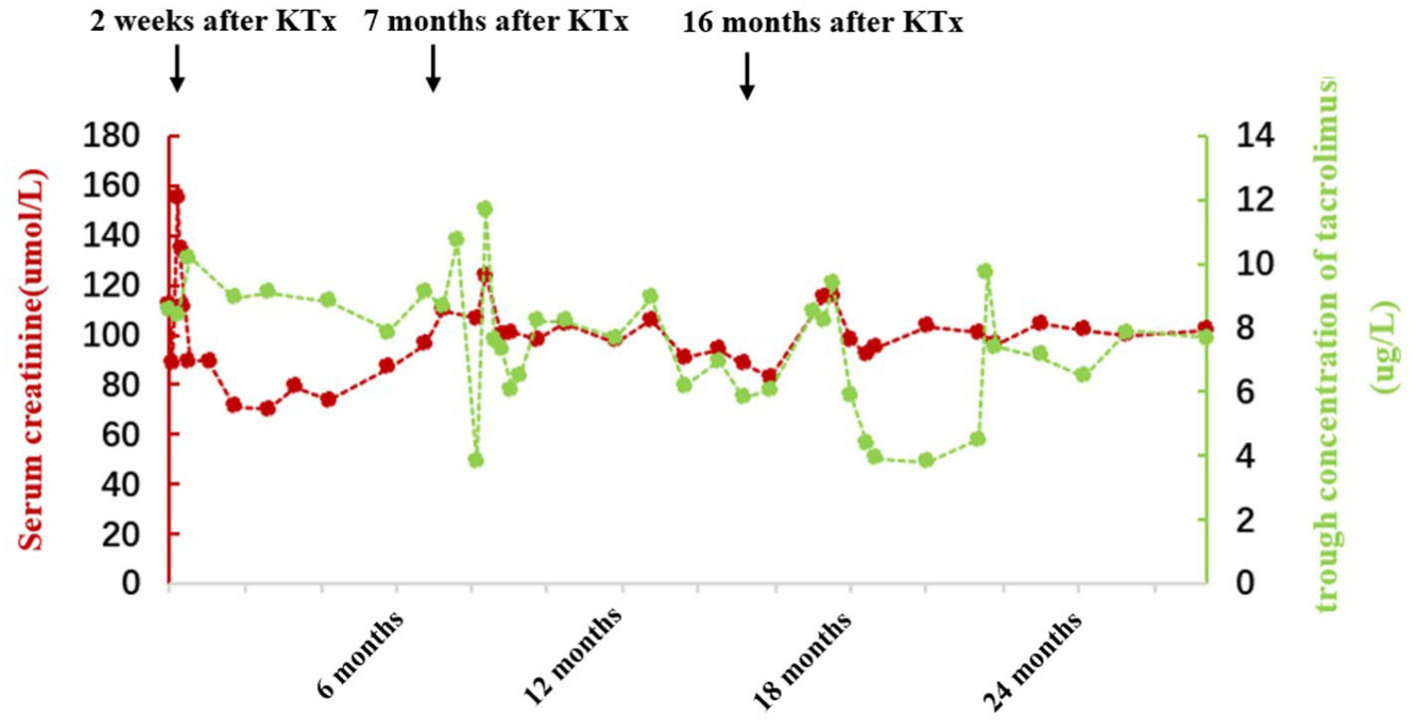
Follow-up of serum creatinine and tacrolimus concentrations after the second KTx in case 2.

Case 2 was a 6-year-old girl who developed fatigue, anorexia, and foamy urine and was diagnosed with SRNS in a local hospital due to glucocorticoid unresponsiveness. The patient progressed to ESRD at 7 years of age and was continued on hemodialysis until she received KTx at the age of 12 years. After KTx, her sCr returned to a normal level, and antirejection therapy of mycophenolate mofetil, prednisone, and tacrolimus was initiated. However, she developed fever, vomiting, and diarrhea in May 2018 (47 months after the first KTx) with a markedly increased sCr level of 458 μmol/L and a tacrolimus trough concentration of 6.1 ng/ml. The patient then received the first renal graft biopsy in November 2018 (53 months after the first KTx), and chronic tubulointerstitial nephritis and chronic allograft rejection (interstitial sclerosis) were indicated (Figures 1A,B). Immunofluorescence on the biopsy section showed immunoglobulin (Ig) G, IgM, IgA, and complements C3 and C1q negative. No segmental glomerular sclerosis was found by light microscope. Serum creatinine continued to increase despite multiple doses of methylprednisolone pulse therapy received at a local hospital. WES was performed in the same year. The patient underwent the second KTx in our hospital on 20 October 2019 due to renal graft failure (64 months after the first KTx). Panel reactive antibodies (PRAs) were positive before the second transplantation (human leukocyte antigen, HLA-I antibody 12%, HLA-II antibody 8%). The same immunosuppressive regimen was prescribed after the second KTx and sCr returned to the normal level. Unfortunately, sCr had risen to 155 μmol/L with an elevated resistance index (RI = 0.82) of kidney transplant 2 weeks after the second KTx. Acute rejection was considered and treatment with anti-thymocyte globulin and methylprednisolone was given. Although sCr returned to a normal level (sCr 70–90 μmol/L) after antirejection treatment, it increased again (sCr 107–124 μmol/L) 7 months later. Therefore, the second graft biopsy was performed on 14 June 2020. Pathological findings showed slight glomerulonephritis and peritubular capillary vasculitis, along with acute renal tubular injury and special epithelial nuclear morphological changes (Figures 1C,D). Complement C4d on the peritubular capillaries, IgG, IgM, IgA, and complements C3 and C1q were all negative. SV40-T and CMV were not detected in renal tubular epithelial cells by immunohistochemistry. Therefore, the dosage of tacrolimus was reduced, and sCr gradually returned to 80–90 μmol/L. After 16 months of the second KTx, we decided to increase the dose of tacrolimus due to weight gain in the patient and low trough concentration (5.8 μg/L). Yet, she could not tolerate the increased dose (sCr increased to 115–116 μmol/L), and therefore the dose of tacrolimus was then lowered to maintain sCr within the normal range (90–100 μmol/L). Urinary protein was negative throughout the follow-up period, and renal function was normal at the last visit. Detailed follow-up data are shown in Figure 2. The patient took medicine regularly with good adherence.

Case 3 was the younger sister of case 2 reported in this study. The patient underwent urinalysis due to her sister's illness, and positive urinary protein (3+) was found. She was also diagnosed with SRNS due to glucocorticoid unresponsiveness at a local hospital. A renal biopsy was not performed, and she progressed to ESRD at the age of 15. KTx was performed in our hospital on 15 January 2021. After KTx, immunosuppressants including tacrolimus and mycophenolate mofetil were prescribed to prevent graft rejection. There was no recurrence in the first year of follow-up with normal urinalysis and renal function (sCr 49 μmol/L). Owing to short stature diagnosed at the age of 12 (Ht 118.5 cm, <-3 SD), the patient was treated with recombinant human growth hormone (rhGH) in another hospital for about 2 years prior to KTx with an increment of 4.5 cm in height per year. The use of rhGH was discontinued due to activation of the gonadal axis, and she had grown 5 cm within 6 months after KTx. Ultrasound showed a thyroid cyst (TR1) but the thyroid function was normal.

Case 4 was an 8-year-old boy who had elevated sCr (430 μmol/L), hypertension (150/110 mmHg), and positive urinary protein (2+) during routine checkups. Antihypertensive medications were administered and trio WES was performed. He progressed to ESRD 1 year later and started peritoneal dialysis. He is still receiving maintenance peritoneal dialysis and waiting for KTx.

**Figure 3:**
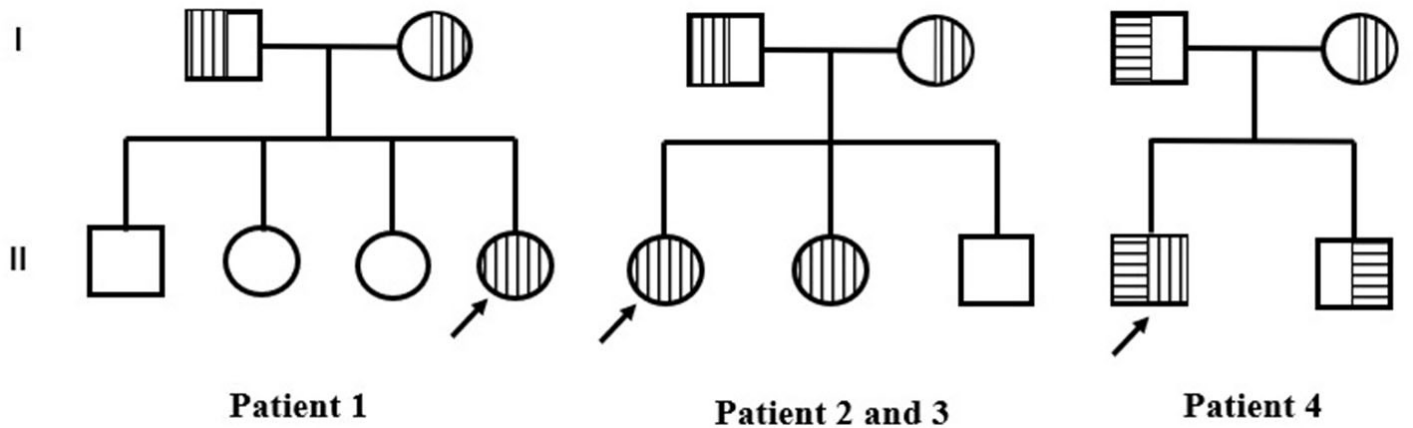
Pedigrees of three families. Empty square and circle show healthy men and women. Vertical stripes represent patients with c.748G>C variants. Horizontal stripes represent patients with c.448C>T. The arrow represents the proband.

Physical examination of all cases did not observe psychomotor retardation or notable abnormalities with eyes or ears. More details are shown in Table 1, and pedigrees are shown in Figure 3. The genetic information indicated that COQ8B-GN caused by *COQ8B* gene mutation was autosomal recessive.

### Results from the literature review

There had been 28 cases of COQ8B-GN who progressed to ESRD and received KTx reported globally. Cases without data on follow-up and patient outcomes were excluded (1, 12–14). A total of 18 cases were included for subsequent analysis (15–19). The median follow-up time was 24 (IQR 17.8–95.0) months, up to 180 months. Except for two cases that developed graft failure caused by hyperacute rejection (HAR) and chronic rejection, respectively, graft function was good without recurrence in all other cases (Table 2).

**Table 2:** Published cases of kidney transplantation with COQ8B-GN.

**Case series**	***N***	**Gender**	**Age at onset (years)**	**Renal manifestation**	**Pathological finding**	**Extrarenal manifestation**	**Nucleotide alteration**	**Age at ESRD (years)**	**Age at KTx (years)**	**Follow-up after KTx (months)**	**Outcome**
Atmaca et al. (15)	7	F	13	P, CKD	ND	None	c.293T>G(HOM)	13	13	87	Last eGFR:74 ml/min/1.73 m^2^
		M	16.7	NS,ESRD	ND	None	c.1339dupG(HOM)	16.7	16.7	103	Last eGFR:80 ml/min/1.73 m^2^
		F	13	P, CKD	ND	None	c.1199dupA(HOM)	ND	16	76	Graft loss after 5 years due to CAN
		M	5	NS, CKD	ND	None	c.1199dupA(HOM)	ND	13	42	Last eGFR:126 ml/min/1.73 m^2^
		F	20.3	P, ESRD	ND	None	c.1339dupG(HOM)	20.3	20.5	58	Graft loss due to HAR
		F	16.4	NS, CKD	ND	None	c.1339dupG(HOM)	ND	17	12	Last eGFR:120 ml/min/1.73 m^2^
		M	6.4	P, CKD	ND	Seizure	c.1339dupG(HOM)	ND	11	186	Last eGFR:57 ml/min/1.73 m^2^
Song et al. (16)	7	M	6.5	P, CKD5	DMS	None	c.532C>T(Het) c.737G>A(Het)	6.5	ND	Median:17.8 (IQR 15.2–19.7)	All had excellent graft survival although 2 cases had acute rejection
		M	10.2	P, CKD5	None	Macula Retinitis	c.532C>T(Het) c.737G>A(Het)	10.9	ND		
		F	7.6	SRNS	FSGS	None	c.737G>A(HOM)	7.7	ND		
		F	3.6	P	FSGS	None	c.737G>A(Het) c.936-938delGGT (Het)	9.4	ND		
		F	9.8	SRNS	MsPGN	Seizure	c.748G>C (HOM)	9.8	ND		
		M	3.3	SRNS	ND	Arrhythmia	c.748G>C(HOM)	3.9	ND		
		F	11.1	P	ND	Seizure	c.748G>C(Het) c.1468C>T(Het)	11.1	ND		
Adán Lanceta V et al. (17)	1	M	5.5	P, ESRD	ND	PMR, RP Febrile seizures	c.439T>C(Het) c.1035+2T>C(Het)	6	ND	ND, aged 12 years old at last visit	Normal renal graft function
Fareed et al. (19)	1	F	21	Renal dysfunction	ND	None	c.748G>A(HOM)	ND	23	24	Normal renal graft function
Wang et al. (18)	2	M	7	P	MsPGN	None	c.532C>T(Het) c.748 G>C(Het)	14	16	132	Normal urinalysis and renal graft function
		F	10	SRNS	FSGS	None	c.532C>T(Het) c.748 G>C(Het)	13	14	180	Normal urinalysis and renal graft function

ND, no data or no done; HOM, homozygous; het, heterozygous; F, female; M, male; CKD, chronic kidney disease; P, proteinuria; NS, nephrotic syndrome; ESRD, end-stage renal disease; CAN, chronic allograft nephropathy; HAR, hyperacute rejection; KTx, kidney transplantation; DMS, diffuse mesangial sclerosis; FSGS, focal segmental glomerulosclerosis; SRNS, steroid-resistant nephrotic syndrome; MsPGN, mesangial proliferation glomerulonephritis; PMR, psychomotor retardation; RP, retinitis pigment.

**Figure 4:**
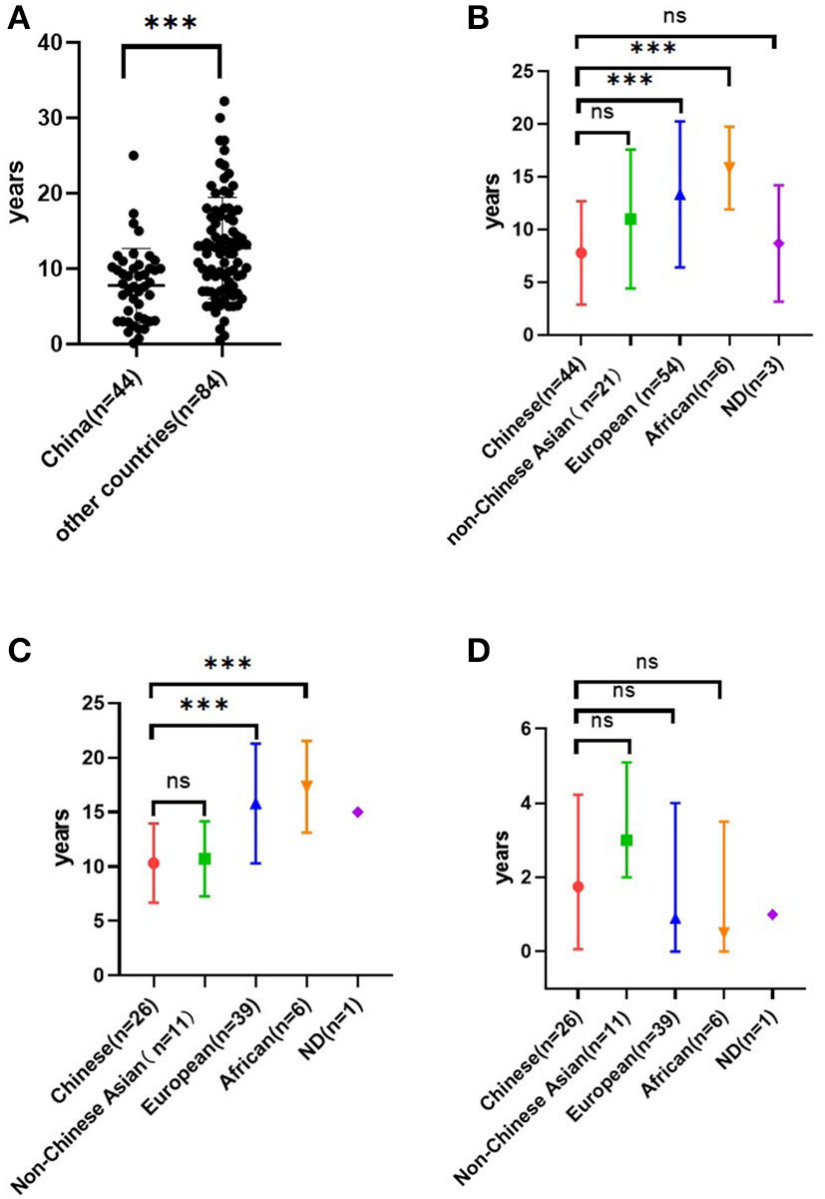
Age of onset in COQ8B-GN among the Chinese population and the non-Chinese population based on published literature **(A)**. Age of onset in COQ8B-GN among different ethnic origins. Non-Chinese Asians included Indians (*n* = 3), Koreans (*n* = 14), Arabs (*n* = 2), and Japanese (*n* = 2). European (*n* = 54) included Turkish, French, German, Belgian, and Spaniard. African included Tunisian (*n* = 2), Moroccan (*n* = 2), and Algerian (*n* = 2). ND, no data **(B)**. The age of ESRD in COQ8B-GN among different ethnic origins **(C)**. Time from onset to ESRD in COQ8B-GN among different ethnic origins **(D)**. ***P < 0.01. ns, no significance.

There were 46 cases of COQ8B-GN among the Chinese population published to date. There were 44 cases left after two patients with *NPHS1* homozygous mutation (20) were excluded (5, 6, 12, 16, 18, 21–24). Of these cases, 75% (33/44) had their onset before or at the age of 10 years (range 10 days to 25 years). The median age of onset in the Chinese population was earlier than that of non-Chinese patients (1, 13–15, 17, 19, 25–29) (Figure 4A). We also found that the age of onset in the Chinese population was younger than that in other Asian populations, but the difference was not significant (*P* = 0.051; Figure 4B). Published data also demonstrated that Asian patients with COQ8B-GN progressed to ESRD earlier than non-Asian patients (Figure 4C). However, the duration from onset to ESRD did not differ among ethnic groups (Figure 4D). COQ8B-GN had insidious onset with various clinical manifestations, including asymptomatic proteinuria, nephrotic syndrome (NS), or SRNS. Extrarenal involvements were uncommon and only seen in nine reported cases, and symptoms included seizure (*n* = 2), brain development retardation (*n* = 1), arrhythmia (*n* = 1), vesicoureteral reflux (*n* = 1), ovarian cyst (*n* = 1), macula retinitis (*n* = 1), low serum C3 level (*n* = 1), and cataract (*n* = 1). Notably, the case complicated with brain development retardation might be caused by a combination of other gene mutations (such as *ARHGEF6, ARID1A*, and *SETBP1*). We were not able to rule out if the cataract was related to the use of glucocorticoids. By the time of literature publication, 59% (26/44) of patients had progressed to ESRD and required dialysis or renal transplantation. The median time from onset to ESRD was 21 (IQR 0.75–50.75) months. The mean age at the time of ESRD diagnosis was 10.3 ± 3.6 years. The pathological findings of COQ8B-GN in the Chinese population varied. Among the reported cases, 34 underwent renal biopsy, most of which were diagnosed with FSGS (61.8%, 21/34), followed by mesangial proliferative glomerulonephritis (20.6%, 7/34). Other pathological findings included endocapillary proliferative glomerulonephritis, 100% glomerular obsolescence, diffuse mesangial sclerosis, minimal change disease, sclerosing glomerulonephritis, and mitochondrial nephropathy. The most prevalent mutation in the *COQ8B* gene among the Chinese patients with COQ8B-GN was c. 748G>C, followed by c.737G>A and c.532C>T (Supplementary Table 1). Moreover, we found that c.737G>A and c.1339dupG were the most frequent mutations in the *COQ8B* gene among non-Chinese Asians and Europeans, respectively.

## Discussion

The *COQ8B* gene is located on chromosome 19q13.2 and encodes a protein with a protein kinase domain. In 2013, Ashraf et al. (1) identified *COQ8B* (*ADCK4*) as one of the pathogenic genes of SRNS for the first time by combining techniques of homozygosity mapping (HM) and whole-exome resequencing (WER). More cases have been reported worldwide since then. According to the existing reports, ethnic differences in detection rates of the *COQ8B* mutation among SRNS or FSGS patients emerged, despite the rarity of the disease. The reported *COQ8B* mutation detection rate in the Chinese population was 5.7%−6.7% (6, 30), which was higher than that in the Korean population (2.7%) (29) and other Western countries (France, Turkey, and Germany, 4.9%) (25). According to some non-Chinese case reports (1, 15, 25), *COQ8B gene* mutation causes adolescence-onset nephropathy. However, all patients in this study presented initial symptoms before the age of 10, and three of them had progressed to ESRD before puberty. Current published data also support that Chinese patients developed COQ8B-GN earlier than non-Chinese patients (Figure 4A), which might be related to genotype–phenotype correlations. Considering the insidious onset and rapid progression to ESRD, routine urinalysis is important to detect the disease at an early stage. For those with unexplained proteinuria or SRNS, it is recommended to conduct genetic testing as early as possible to assist in diagnosis, which can avoid unnecessary use of steroids or other immunosuppressants. Besides, COQ8B-GN is an autosomal recessive disorder. In our study, case 3 underwent urinalysis and genetic sequencing due to positive family history, emphasizing the importance of a thorough assessment of the patient's family history. We also noticed that the age of onset in patients with COQ8B-GN ranged widely from 10 days to 25 years in the Chinese population and from <1 year to 32.2 years in the non-Chinese population. Hence, even in adult-onset proteinuria, COQ8B-GN should be suspected. Since CoQ10 plays an important role in cell metabolism, its synthesis defect can affect various organs, resulting in various clinical manifestations, especially those with high energy demands including the brain, muscles, and kidneys (31). However, extrarenal involvement was infrequently observed in COQ8B-GN, which might be related to the enrichment of COQ8B in podocytes. Neurological dysfunction, such as seizure and intellectual disability, was the most commonly encountered extrarenal involvement (15, 17, 25), followed by cardiovascular system involvement, such as hypertrophic cardiomyopathy, arrhythmia, and cardiac insufficiency. Retinitis pigmentosa, hypothyroidism, and Crohn's disease had also been reported in the past (15, 25, 26). In this study, only one case was complicated with a thyroid cyst, which has not been reported before. Treatment with ubiquinone could reduce proteinuria in asymptomatic patients (4) and improve psychomotor development (17). Thus, careful physical examination is necessary to help exclude extrarenal involvement in patients with *COQ8B* mutation. Early intervention with CoQ10 supplementation should be started before irreversible organ damage. However, all four cases in this study did not receive CoQ10 supplementation since they had already progressed to ESRD at the time of genetic diagnosis.

Similar to other podocyte diseases caused by gene mutations such as *NPHS1, NPHS2*, and *WT1* (32), FSGS is the most prevalent histological change in COQ8B-GN, followed by mesangial proliferative glomerulonephritis among the Chinese population (Supplementary Table 1). Sclerosing glomerulonephritis, mitochondrial nephropathy, and glomerular minimal change have also been reported domestically and internationally, indicating the heterogeneity of renal pathology in COQ8B-GN.

Genetic nephrotic syndrome is usually associated with immunosuppressant unresponsiveness and rapid progression to ESRD. Renal transplantation was an effective treatment with a low risk of post-transplantation recurrence in NS of genetic causes. However, it has been reported that recurrence occurred in patients with homozygous Fin-major mutation in *NPHS1* after transplantation, which was caused by total loss of nephrin protein without immune tolerance. After KTx, these patients produced anti-nephrin antibodies, causing recurrence and graft failure (33). Francis et al. (34) found that the recurrence rate in recipients with non-secondary FSGS reached 10.3%, which usually occurred within 2 years after KTx. However, summative research on the recurrence risk of COQ8B-GN after KTx is still lacking. We summarized the evidence derived from previous research (Table 2), suggesting that the recurrence risk is low in patients with COQ8B-GN after KTx. Particularly, renal graft biopsy was performed two times in case 3 and no sign of FSGS was shown, which further strengthened this viewpoint. By integrating the previous and present data, there were two cases of acute rejection, two cases of chronic rejection, and 1 case of hyperacute rejection reported, and three of these cases developed graft failure, indicating that rejection remains an important threat to long-term graft survival.

In addition, there are more challenges in the management of children after KTx (35). First, young infants have relatively immature immune systems, putting them at a high risk of infection such as polyomavirus and causing allograft dysfunction. Second, other factors relevant to adolescence, such as poor medication adherence and enhanced immune potency, can also give rise to rejection. In case 2 reported, the trough concentration of tacrolimus fluctuated greatly during follow-up (Figure 2), but she denied missing or self-stopping the medication. In fact, besides medication adherence, other factors such as diarrhea, the timing of immunosuppressant administration, and interactions between medications and food also contribute to intra-patient variability (IPV). High IPV is associated with poor graft survival, a higher risk of acute rejection, histologic lesions, and the production of donor-specific antibodies (36). Due to the wide variability in interpatient pharmacokinetics and narrow therapeutic index of tacrolimus, close monitoring of drug concentration and dosage adjustment is needed.

In this study, two cases were complicated by short stature. Growth retardation is common among children with CKD and it accounts for up to 12.1% of the initial manifestation of CKD (37). Conversely, short stature is also associated with an increased risk of death (38) and psychosocial stress, which deserves our serious attention. Some pediatric patients can obtain catch-up growth after KTx by improving metabolic and endocrine disturbances, as in Case 3 reported in our study, and in those who discontinue glucocorticoids before puberty or soon after transplantation (39). For those who have persistent growth failure even if the potentially treatable risk factors have been adequately addressed or for those who do not have spontaneous catch-up growth after KTx, rhGH should be considered (9).

## Conclusion

In conclusion, our results show that COQ8B-GN in the Chinese population often develops symptoms of asymptomatic proteinuria or SRNS before the age of 10 years. FSGS is the most prevalent pathological finding, and c.748G>C variant of the *COQ8B* gene is the most frequent genetic mutation in Chinese patients. More than half of the Chinese patients would progress to ESRD at diagnosis or within 2 years of diagnosis during the period of early adolescence. Despite the risk of allograft rejection, renal transplantation is feasible for COQ8B-GN patients with ESRD due to the low risk of recurrence.

## Data availability statement

The original contributions presented in the study are included in the article/Supplementary materials, further inquiries can be directed to the corresponding authors.

## Ethics statement

The studies involving human participants were reviewed and approved by the Ethics Committee of the First Affiliated Hospital, Sun Yat-sen University. Written informed consent to participate in this study was provided by the participants' legal guardian/next of kin. Written informed consent was obtained from the minor(s)' legal guardian/next of kin for the publication of any potentially identifiable images or data included in this article.

## Author contributions

SZ, YX, and CC conducted the review of the literature and wrote the first draft of the manuscript. XJ and LC designed the study, reviewed, and revised the manuscript. NY, LL, and YM collected, analyzed, interpreted clinical, imaging, genetic data of four cases, and contributed to manuscript revision. All authors contributed to the manuscript and approved the final version.

## Funding

This study was supported by the Science and Technology Planning Project of Guangzhou, China (Grant No. 202103000001).

## Conflict of interest

The authors declare that the research was conducted in the absence of any commercial or financial relationships that could be construed as a potential conflict of interest.

## Publisher's note

All claims expressed in this article are solely those of the authors and do not necessarily represent those of their affiliated organizations, or those of the publisher, the editors and the reviewers. Any product that may be evaluated in this article, or claim that may be made by its manufacturer, is not guaranteed or endorsed by the publisher.
