# Supplementary material for: COQ8B glomerular nephropathy: Outcomes after kidney transplantation and analysis of characteristics in Chinese population
Source: Front Pediatr. 2022 Aug 10;10:938863. doi: 10.3389/fped.2022.938863 (PMC9399612; doi:10.3389/fped.2022.938863)
Supplement: Supplementary file 1 [file Table_1.DOCX]

**Supplemental Table 1**.Clinical features, genotypes and prognosis of COQ8B-GN in Chinese population based on published literature.

| **Case series** | **N** | **Gender** | **Age at onset (years)** | **Initial**  **presentation** | **Histology** | **Extrarenal manifestations** | **Nucleotide alteration** | **Age at ESRD**  **(years)** | **Time from onset to ESRD(months)** | **Treatment** | **Outcome** |
| --- | --- | --- | --- | --- | --- | --- | --- | --- | --- | --- | --- |
| Song X et al,2017 | 8 | F | 9 | P | FSGS | None | c.737G>A(Het)  c.748G>C(Het) | - | - | GC,TAC partial response ACEI,CoQ10 | CKD 2 |
|  |  | M | 6.5 | SRNS | GO(100%） | None | c.737G>A(HOM) | 6.5 | 0 | PD, CoQ10 | CKD 5 |
|  |  | F | 7.6 | SRNS | FSGS | None | c.737G>A(HOM) | 7.6 | 0 | PD, CoQ10 | KTx at 8.2 years old |
|  |  | M | 2 | SRNS | MsPGN | None | c.748G>C(HOM) | - | - | CoQ10,No response to TAC,MMF | CKD2 |
|  |  | F | 9.8 | SRNS | EPGN | None | c.748G>C(HOM) | 9.8 | 0 | PD, CoQ10 | CKD 5 |
|  |  | F | 11.7 | P | FSGS | None | c.748G>C(HOM) | 13.7 | 24 | ACEI, CoQ10,PD | CKD 5 |
|  |  | M | 3 | P | FSGS | None | c.551A>G(Het)  c.737G>A(Het) | 8 | 60 | PD, CoQ10;No response to GC,CSA,TAC,CTX or Rituximab | CKD 5 |
|  |  | F | 3 | P | FSGS | None | c.737G>A(HOM) | - | - | CoQ10, No response to GC | CKD 1 |
| Wang F et al,2017 | 8 | M | 11 | P | FSGS | Low serum  C3 level | c.241G>T(Het)  c.1468C>T(Het) | - | - | No response to GC | Normal renal function |
|  |  | F | 8 | SRNS | FSGS | ND | c.448C>T(Het)  c.748G>C(Het) | 11.7 | 44 | ND | ESRD |
|  |  | F | 9.2 | SRNS | Sclerosing  glomerulonephritis | ND | c.532C>T(Het)  c.748G>C(Het) | 11 | 22 | ND | ESRD |
|  |  | F | 8.2 | SRNS | FSGS | ND | c.737G>A(HOM) | - | - | ND | Normal renal function |
|  |  | F | 17.3 | P | MsPGN | ND | c.737G>A(HOM) | - | - | ND | Normal renal function |
|  |  | F | 10 days | NS | ND | ND | c.748G>C(HOM) | ND | ND | ND | ND |
|  |  | F | 1.6 | SRNS | FSGS | ND | c.748G>C(HOM) | 6 | 53 | ND | ESRD |
| **Case series** | **N** | **Gender** | **Age at onset (years)** | **Initial**  **presentation** | **Histology** | **Extrarenal manifestations** | **Nucleotide alteration** | **Age at ESRD**  **(years)** | **Time from onset to ESRD(months)** | **Treatment** | **Outcome** |
|  |  | F | 6 | P | FSGS | ND | c.748G>C(Het)  c.1093C>G(Het) | - | - | ND | Normal renal function |
| Feng C et al ,2017 | 2 | F | 9 months | p | ND | MRI:brain development retardation^a^ | c.748G>C(Het)  c.532C>T(Het) | - | - | CoQ10 | Normal renal function  negative urine protein |
|  |  | F^b^ | 9 | SRNS | FSGS | None | c.625C>G(Het)  c.614C>T(Het) | - | - | No response to TAC  CoQ10 | Increased SCr  (80-90umol/L) |
| Yang J et al , 2018 | 1 | M | 10 | p | FSGS | None | c.625C>G(Het)  c.918G>T(Het) | - | - | No response to GC  CoQ10 | UPCR was reduced  Normal renal function |
| Yang Z et al,2019 | 1 | M | 12 | P | MCD | None | c.748C>G(Het)  c.1041G>T(Het) | - | - | No response to GC | CKD 1 |
| Song X et al,2020 | 20 | F | 10 | P | ND | VUR | c.449G>A(HOM) | 11 | 12 | PD | ESRD |
|  |  | M | 2.4 | P | ND | None | c.449G>A(Het)  c.737G>A(Het) | 8 | 67 | PD | ESRD |
|  |  | F | 4.4 | P | FSGS | Ovarian cyst | c.538C>T(HOM) | 11.5 | 85 | No response to GC,TAC  PD | ESRD |
|  |  | M | 2.9 | p | FSGS | None | c.551A>G(Het)  c.737G>A(Het) | 3.1 | 2 | PD | ESRD |
|  |  | F | 3.1 | SRNS | FSGS | None | c.737G>A(HOM) | - | - | ACEI, CoQ10 | ND |
|  |  | F | 9.3 | P | FSGS | None | c.737G>A(Het)  c.748G>C(Het) | 13.5 | 50 | No response to GC;  ACEI,CoQ10,PD | ESRD |
|  |  | M | 7 | SRNS | MsPGN | Short stature | c.737G>A(Het)  c.748G>C(Het) | - | - | ACEI, CoQ10 | ND |
| **Case series** | **N** | **Gender** | **Age at onset (years)** | **Initial**  **presentation** | **Histology** | **Extrarenal manifestations** | **Nucleotide alteration** | **Age at ESRD**  **(years)** | **Time from onset to ESRD(months)** | **Treatment** | **Outcome** |
|  |  | M | 2 | SRNS | MsPGN | Cataract | c.748G>C(HOM) | - | - | No response to GC,TAC;  ACEI, CoQ10 | ND |
|  |  | F | 10.5 | P | ND | None | c.748G>C(HOM) | 12.2 | 20 | CoQ10, PD | ESRD |
|  |  | M | 5.3 | P | FSGS | None | c.748G>C(HOM) | - | - | ACEI, CoQ10 | ND |
|  |  | F | 11.7 | P | MsPGN | None | c.748G>C(HOM) | 13.9 | 26 | ACEI,CoQ10,PD | ESRD |
|  |  | M | 15 | P | FSGS | None | c.893+2T>A(Het)  c.1035+3A>G(Het) | 17.2 | 26 | HD | ESRD |
|  |  | M | 16 | P | ND | None | c.770+2T>A(Het)  c.912+3A>G(Het) | 17.6 | 19 | HD | ESRD |
|  |  | F | 7.6 | SRNS | FSGS | None | c.737G>A(HOM) | 7.7 | 1 | PD-KTx | All had great graft survival without recurrence of NS or proteinuria although two developed acute rejection. |
|  |  | M | 6.5 | P | DMS | None | c.532C>T(Het)  c.737G>A(Het) | 6.5 | 0 | PD-KTx |  |
|  |  | M | 10.2 | P | ND | Macula Retinitis | c.532C>T(Het)  c.737G>A(Het) | 10.9 | 8 | PD-KTx |  |
|  |  | F | 3.6 | P | FSGS | None | c.737G>A(Het)  c.936-938delGGT  (Het) | 9.4 | 70 | No response to GC,TAC  PD-KTx |  |
|  |  | F | 9.8 | SRNS | MsPGN | Seizure | c.748G>C(HOM) | 9.8 | 0 | No response to GC,TAC  PD-KTx |  |
|  |  | M | 3.3 | SRNS | ND | Arrhythmia | c.748G>C(HOM) | 3.9 | 7 | No response to GC,TAC  ACEI, CoQ10, PD-KTx |  |
|  |  | F | 11.1 | P | ND | Seizure | c.748G>C(Het)  c.1468C>T(Het) | 11.1 | 0 | PD-KTx |  |
| **Case series** | **N** | **Gender** | **Age at onset (years)** | **Initial**  **presentation** | **Histology** | **Extrarenal manifestations** | **Nucleotide alteration** | **Age at ESRD**  **(years)** | **Time from onset to ESRD(months)** | **Treatment** | **Outcome** |
| Zhai SB et al,2020 | 1 | M | 3 | P | Mitochondrial nephropathy | None | c.(271C>T) (Het)  c.(737G>A)(Het) | - | - | ACEI, CoQ10 | Normal eGFR  Proteinuria decreased |
| Zhang Y et al,2021 | 1 | F | 25 | P | ND | None | c.737G > A(Het)  c.577-600del(Het) | - | - | ACEI, CoQ10 | eGFR remained stable  （50-60ml/min） |
| Wang S et al,2022 | 2 | M | 7 | SRNS | MsPGN | None | c.532 C>T(Het)  c.748 G>C(Het) | 14 | 84 | KTx | Normal urinalysis and renal graft function |
|  |  | F | 10 | SRNS | FSGS | None | c.532 C>T(Het)  c.748 G>C(Het) | 13 | 36 | KTx | Normal urinalysis and renal graft function |

HOM: homozygous;Het, heterozygous; P:proteinuria;F:female; M:male;FSGS:focal segmental glomerulosclerosis;GO: glomerular obsolescence;SRNS:steroid-resistant nephrotic syndrome; MsPGN:mesangial proliferation glomerulonephritis;CKD:chronic kidney disease;GC: glucocorticoid;TAC: Tacrolimus;PD: peritoneal dialysis; KTx;kidney transplant;MMF: mycophenolate mofetil; EPGN: endocapillary proliferative glomerulonephritis; CSA: Ciclosporin;CTX: cyclophosphamide;MCD: minimal change disease;ND：no data or not done; ESRD: end-stage renal disease;SCr:serum creatinine; UPCR:urinary protein/creatinine ratio; VUR:vesicoureteral reflux;DMS:diffuse mesangial sclerosis.

^a^: brain developmental retardation of this patient may be due to other genetic mutations such as *ARHGEF6*,*ARID1A* and *SETBP1* genes.

^b^: This patient also carried heterozygous mutations in *NPHS1* gene(c.1802C>G and c.1339C>T) but her father showed no signs for nephropathy with the same genotype in *NPHS1 gene*
